# Sixteen-year predictors of successful aging from a Southern Brazilian cohort The PALA study

**DOI:** 10.1590/1980-57642018dn12-030002

**Published:** 2018

**Authors:** Juciclara Rinaldi, Gabriele do Canto Souza, Ana Luiza Camozzato, Márcia Lorena Fagundes Chaves

**Affiliations:** 1 Dementia Clinic, Neurology Service, Hospital de Clínicas de Porto Alegre, Porto Alegre, RS, Brazil.; 2 Federal University of Health Sciences of Porto Alegre, RS, Brazil.; 3 Internal Medicine Department, UFRGS School of Medicine, Porto Alegre, RS, Brazil.

**Keywords:** successful aging, predictors, cohort, aging, envelhecimento bem-sucedido, preditores, coorte, envelhecimento

## Abstract

Successful aging (SA) is a current positive aging-related perspective and it is important to determine the variables associated with this concept. Most longitudinal population-based studies on predictors of SA were carried out in developed countries.

**Objective:**

This investigation evaluated which baseline characteristics predicted successful aging in 16 years of follow-up in a southern Brazilian cohort - the Porto Alegre Longitudinal Aging study.

**Methods:**

At baseline, 345 community-dwelling healthy independent individuals aged 60 or older were assessed for medical and psychiatric conditions, memory, orientation, judgment and problem solving, functioning in the community and at home, and hobbies. SA, according to Rowe and Kahn’s definition, was the outcome assessed in the last evaluation at a maximum 16-year follow-up. All baseline variables were evaluated as potential predictors for the outcome SA.

**Results:**

Of the 345 individuals evaluated at baseline, 32 (9.3%) participants were classified as successful agers in the follow-up. Younger age (OR=0.926, 95%CI=0.863-0.994), female gender (OR=0.226, 95%CI=0.072-0.711) and higher MMSE (OR=1.220, 95%CI=1.031-1.444) were predictors of SA for the 16-year follow-up in a logistic regression model.

**Conclusion:**

In contrast with our previous hypothesis, the impact of the socioeconomic and socio-environmental characteristics was small, as was the baseline classification into successful and normal aging.

## RESUMO

Envelhecimento bem sucedido (EBS) é uma perspectiva positiva sobre o envelhecimento, sendo importante determinar as variáveis associadas a esse conceito. A maioria dos estudos longitudinais de base populacional sobre preditores do EBS foi realizada em países desenvolvidos.

**Objetivo:**

Este estudo avaliou quais características da linha de base predisseram o envelhecimento bem sucedido em 16 anos de seguimento de uma coorte do sul-brasileira - o Estudo Longitudinal de envelhecimento de Porto Alegre (*Porto Alegre Longitudinal Aging - PALA - study*).

**Métodos:**

No início do estudo, 345 indivíduos da comunidade, saudáveis e independentes, com 60 anos ou mais foram avaliados para condições médicas e psiquiátricas, memória, orientação, julgamento e solução de problemas, funcionamento na comunidade, em casa e passatempos. EBS, segundo Rowe e Kahn, foi o desfecho obtido na última visita em um máximo de 16 anos de seguimento. Todas as variáveis da linha de base foram avaliadas como potenciais preditoras para o desfecho EBS.

**Resultados:**

Dos 345 indivíduos avaliados na linha de base, 32 participantes (9,3%) foram classificados como idosos com envelhecimento bem sucedidos em 16 anos de seguimento. No modelo de regressão logística, menor idade (OR=0,926; 95%CI=0,863-0,994), gênero feminino (OR=0,226; 95%CI=0,072-0,711) e MEEM mais elevado (OR=1,220; 95%CI=1,031-1,444) foram preditores de EBS para o seguimento de 16 anos.

**Conclusão:**

Em contraste com nossa hipótese prévia, o impacto das características socioeconômicas e socio-ambientais foi pequeno, bem como o da classificação inicial de envelhecimento normal e bem sucedido.

Not all older adults age at the same rate, or to the same extent. In fact, some older adults do not follow the typical aging profile, attracting increasing interest in what constitutes “successful aging”.^1^ Older individuals who had low probability of disease and disease-related disability, high cognitive and physical functional capacity, active engagement with life, and social and productive functioning were called “successful agers”.^2^^,^ 3 Since its introduction, the term “successful aging” (SA) has been a concept expressing a shift in the focus towards aging, regarding aging no longer as a synonym of disease, but as the possibility of satisfactory functioning, with no disabilities.^2^

This positive aging perspective has prompted a search for variables associated with SA, especially through longitudinal population-based studies. Among these investigations, socioeconomic advantage across all life stages was associated with SA in two Scottish cohorts aged around 57 and 76.^4^ Additionally, in the Manitoba Longitudinal Study of Aging, a representative sample of individuals aged 65-84 living in the community followed for twelve years showed age, four measures of health status, two measures of mental status, and not having one’s spouse dying or institutionalized in nursing homes were shown to be predictors of SA.^5^ The Alameda County Study, another population-based investigation, showed younger age, higher education, white ethnicity, absence of some chronic diseases (diabetes, chronic obstructive pulmonary disease, arthritis and hearing problems, and depression), having close personal contacts and frequent walks for exercise as six-year predictors of SA in subjects aged 65-95 years.^6^ Higher education and younger age were also associated with SA in a Taiwanese older cohort.^7^ The Depp and Jeste review on SA^3^ also pointed to younger age,^8^ health-related variables (such as absence of arthritis, hearing problems),^6^ better activities of daily living,^9^ and not smoking,^8^ as strong predictors of SA.

Most of these longitudinal studies were carried out in developed countries, therefore epidemiological data on predictors of SA derived from developing and poorer countries are scarce. In Brazil, two epidemiological studies evaluated this subject in community-based samples using cross-sectional designs. An association of better friend and family relationships, self-perceived well-being, functional capacity and psychosocial support with successful aging was shown in a sample of 400 older subjects.^10^ A previous study by our research group evaluating baseline data of the PALA (Porto Alegre Longitudinal Aging) study, an ongoing cohort started with 345 community-dwelling healthy independent individuals aged 60 years and older living in southern Brazil classified the participants into normal or successful at the time and found a higher number of living children as a risk factor, whereas confidants and higher family income were protective factors for SA.^11^ Taken into account the cultural and socioeconomic diversity of developing countries, we hypothesized education and income as the strongest predictors of SA in these regions. The inequality of both variables in countries such as Brazil, with wide range distribution, could impact other SA determinants such as health care barriers, poor infrastructure to deal with the needs of the elderly population, and paucity of social network variables and of cognitively enriched environments. Furthermore, we also hypothesized subjects who were classified as successful agers at baseline of this Brazilian cohort would have a higher likelihood of retaining this status during the follow-up. Considering the relevance of this issue and lack of data derived from longitudinal studies conducted in developing countries, the present investigation aimed to evaluate which baseline characteristics predicted successful aging in 16 years of follow-up in this southern Brazilian cohort - the PALA study.

## METHODS

Individuals included in the current study were derived from the Porto Alegre Longitudinal Aging - PALA - study^11^^,^ 12 ^,^ 13 an ongoing longitudinal population-based study on aging.

In 1996, a sample of 345 individuals aged 60-92 living in the community was interviewed at the baseline of a cohort study.^11^^,^ 12 According to data from the 1992 census (IBGE, 2001), 5,500 individuals ≥60 years old resided in the catchment area of the Hospital de Clínicas de Porto Alegre. During a 10-month period in 1996, we visited 2,305 households in that area and selected, according to a multistage random stratification by age, one person ≥60 y.o. per household for a total of 1,216 participants. Of the 1,216 interviewed elders, 369 without cognitive deficit or major medical disorders were selected and 23 (6.3%) refused to participate. The final baseline sample comprised 345 participants. This sample size was sufficient to detect a frequency of 5% cognitive impairment, with an error of 2.5% and a confidence interval (CI) of 95%.^11^ In 2002, 2008 and 2012 they were thoroughly reassessed at their households, and telephone interviews were carried out between these periods.

At baseline, all subjects had to be healthy and independent, aged ≥60 years, and residing in the catchment area of the Hospital de Clínicas de Porto Alegre, in the city of Porto Alegre, southern Brazil. An informant was also used to verify participants’ information. Participants were excluded if they had age-related diseases, psychiatric diagnosis, or disabilities at baseline. All participants and their informants had to report normal functioning in the community at study entry.

Participants were assessed for medical and psychiatric conditions, memory, orientation, judgment and problem solving, functioning in the community and at home, and hobbies. The baseline variables were age, education (in years), gender, family monthly income (in US$), family members with income, children living, siblings living, confidants (yes/no), living with partner, loneliness, number of medical appointments last month, getting help when sick, attending 3^rd^ age groups, the Montgomery-Asberg depression rating scale (MADRS),^14^ the MMSE,^15^ and the SRQ-20, a WHO instrument to screen for general psychiatric disturbances that has been found to be reliable, valid, and adaptable for screening mental disorders in many countries.^16^ Although all individuals scored 0 on the CDR and were functionally independent at study entry, they were further subclassified at baseline into successful agers (defined as good health condition, the complete absence of functional disability and mood disorders, and no cognitive impairment) and normal agers (individuals who had age-determined responses and behaviors without contamination by specific disease processes). Participants from this latter group had minor health problems, some level of cognitive frailty (lower MMSE scores but no impairment), and a small degree of functional disability (lower scores on the Katz ADL scale but no dependence).^11^

The follow-up assessments were composed of demographic, clinical, cognitive and functional data. An interview for sociodemographic and life arrangement information and health conditions, the Mini-Mental State Exam (MMSE);^15^ the CDR scale global score (CDR-GS) and sum of the boxes scores (CDR-SB);^17^^,^ 18 Katz ADL and ADLI scales;^19^ and the Geriatric Depression Scale (GDS)^20^ were the instruments applied. Trained health sciences undergraduate students carried out these evaluations. The vital status of participants was assessed by annual phone calls, and by the thorough follow-up evaluations at each period of home assessments. Additionally, family members were asked to contact the research team by phone when participants had died. Causes and dates of death were also obtained from the official death-certificates.

The outcome variable for this study was successful aging (SA). Successful aging was defined as to be alive, have good health condition, complete absence of functional disability and mood disorders, and no cognitive impairment (CDR-GS=0) in the last thorough assessment. Individuals who did not exhibit these characteristics and those who died during the follow-up were classified as non successful agers. Causes of death as consequence of violence, trauma, or any other that could prevent the opportunity to age successfully were exclusions. The outcome for the study was assessed in the last evaluation at a maximum of 16-year follow-up. All baseline variables were evaluated as potential predictors for the outcome SA.

### Data analyses

The statistical analysis was based on data collected at baseline and from the last follow-up visit (status), during which participants underwent a thorough evaluation. The statistical analysis was performed with the Statistical Package for the Social Sciences (SPSS for Windows 18.0) software. Descriptive data (mean, SD and frequency) were calculated for demographic and clinical data. Parametric and non-parametric data were analyzed with the Student’s *t*-test or Mann-Whitney test, respectively. Categorical variables were tested by the Chi-square test, with Yates correction or the Fisher exact test.

The available baseline variables from the 1996 interview, which could represent potential predictors for the outcome successful aging, were first entered as independent variables in univariate analyses. Thus, the association of demographic data, variables related to care with health, socioeconomic characteristics, social support and social network, with successful aging was evaluated. The baseline classification of successful or normal ager was also assessed as a potential predictor of SA in the follow-up. Categorical variables were tested by the Chi-square test, with Yates correction or the Fisher exact test. Student’s *t-*test or the Mann-Whitney test was used to verify the association of continuous variables and the outcome successful aging. The variables that showed association with successful aging in the univariate models were entered in a binary logistic regression to evaluate their independent effect on this outcome.

The study was approved by the Ethics Committee for Research at the Hospital de Clínicas de Porto Alegre (GPPG #10-0347) and was conducted in accordance with the Declaration of Helsinki. All subjects gave informed written consent.

## RESULTS

Of the 345 individuals evaluated at baseline in 1996, 32 (9.3%) participants were classified as successful agers, and 178 (51.6%) were categorized as non successful agers in the last evaluation at a maximum of 16-year follow-up. Of the 178, only 26 were still alive 16 years later where 152 individuals from the non successful ager group had died during the follow-up. Among the surviving group, those who were classified as SA were predominant. On the other hand, 135 (39.1%) were lost to follow-up. None of the participants died of causes that could prevent the opportunity to age successfully (i.e., car accident, violence etc.). The main causes of death were cardiovascular disease (33%) and cancer (23%), followed by pulmonary disease (17%) and stroke (16%); the other groups were very small or pertained to miscellaneous.

The baseline variables for the evaluated and drop-out groups were compared and the analyses showed significantly higher age (mean±SD 71.6±7.0 and 68.4±6.9, respectively) and lower educational attainment (mean± SD 8.2±4.9 and 10.3±6.1, respectively) in the evaluated group (p<0.001 for both comparisons). No significant difference between the groups was observed for all other baseline variables.

The comparison of baseline characteristics between successful and non-successful agers is shown in Table 1. At baseline, successful agers were younger and had higher cognitive function (measured by MMSE), lower rate of common psychiatric symptoms (measured by the SRQ-20), and no minimal functional disability compared with non-successful agers. Female gender was also associated with SA.

**Table 1:** Comparison of baseline characteristics between successful and non-successful aging groups.

Variables at baseline		Successful (n=32)	Non successful (n=178)	p value
Age (mean, SD)		67.65 (5.33)	72.26 (7.07)	0.001
Gender	Female (N,%)	28 (87.5%)	117 (65.7%)	0.014
Education (mean, SD)		8.41 (3.50)	8.28 (5.14)	0.883
Living with partner	No (N, %)	17 (53.1%)	97 (54.5%)	0.886
Confidant	No (N, %)	5 (15.6%)	42 (23.6%)	0.426
Number of confidants	(mean, SD)	2.22 (1.75)	2.29 (2.84)	0.896
Family Income (monthly minimum wages) (mean, SD)		15.16 (12.83)	22.77 (30.60)	0.175
Number of persons with income (mean, SD)		1.81 (0.78)	2.01 (1.34)	0.416
MADRS (mean, SD)		5.75 (6.16)	7.02 (6.11)	0.280
SRQ-20 (mean, SD)		2.53 (2.65)	3.85 (3.08)	0.023
MMSE (mean, SD)		27.03 (2.42)	24.80 (3.94)	0.002
Loneliness	Never/rarely (N, %)	21 (65.6%)	121 (64%)	0.861
Medical appointments last month (mean, SD)		0.59 (0.80)	0.92 (1.59)	0.255
Adult children living (mean, SD)		2.25 (1.46)	2.63 (2.05)	0.311
Siblings living (mean, SD)		3.19 (2.40)	2.68 (2.83)	0.346
Attend 3^rd^ age groups	No (N, %)	21 (65.6%)	130 (73%)	0.391
Get help when sick	No (N, %)	6 (18.8%)	51 (28.7%)	0.246
Minimal functional disability	Yes (N, %)	0	12 (12.2%)	0.037
Successful ager	Yes (N, %)	22 (68.8%)	108 (57.1%)	0.217

MADRS: Montgomery-Asberg Depression Rating Scale); SRQ-20: Self-Reporting Questionnaire; MMSE: Mini-Mental State Examination.

The baseline variables showing significant difference between groups (age, gender, MMSE, SRQ-20, minimal functional disability) were evaluated in a logistic regression model for the outcome “successful aging” (Table 2). Younger age, female gender and higher MMSE at baseline retained significance and were predictors of SA for the 16-year follow-up.

**Table 2:** Baseline predictors of successful aging during 16-year follow-up: multivariate logistic regression.

Variables	B	S.E.	P value	OR (95% CI)
Sex (male)	–1.488	0.585	0.011	0.226 (0.072-0.711)
Age	–0.077	0.036	0.035	0.926 (0.863-0.994)
MMSE	0.199	0.086	0.021	1.220 (1.031-1.444)
Minimal functional disability	–18.444	7493.99	0.998	0.00 (0.00)
SRQ-20	–0.153	0.082	0.060	0.858 (0. 731-1.006)

SRQ-20: Self-Reporting Questionnaire; MMSE: Mini-Mental State Examination.

## DISCUSSION

In this study we evaluated baseline variables associated with successful aging in community elderly participants from the PALA study after 16 years of follow-up. Demographic, clinical, social support, functional and cognitive information were compared between the groups (successful and non-successful). The successful agers after 16 years of follow-up comprised 32 participants, 9.3% of the original cohort. Because our original sample was composed of only healthy independent elderly individuals, we supposed they would have higher rates of SA in comparison with those derived from a more heterogeneous sample including diseased and functionally impaired older subjects; however, this hypothesis was refuted. The point prevalence of successful aging using Rowe and Kahn’s definition, in a large US sample based on data derived from the Health and Retirement Study for adults aged 65 years and older, was no greater than 11.9% at any of the four time points (1998, 2000, 2002 and 2004) and the odds of SA declined by 25% between 1998 and 2004.^21^ The authors argued that the relevant, but strict and rigorous, Rowe and Kahn’s SA definition, provided the small SA rates. Other authors also pointed to the need of expanding the concepts of successful aging as old individuals get older.^22^ We used the Rowe and Kahn criteria and the use of this definition may have at least partially impacted the lower frequency of SA found in our sample. However, we believe the longer follow-up might be one of the main reasons for this finding. Age itself has been reported as one of the main determinants of SA in several studies^3^^,^ 6 ^-^ 8 and in this lengthy longitudinal observation, all participants were near or above their eighties in the last evaluation. This finding probably increased the chance of these individuals falling into the non-successful aging group since older age is associated with higher prevalence of some chronic diseases and higher mortality.^23^^,^ 24 Other potential contributing factors for the low frequency of SA in our study were the greater frequency of younger and highly educated participants in the drop-out group.

The main goal of the investigation was to evaluate the predictors of SA in a cohort derived from a developing country and we hypothesized that socioeconomic factors would be the strongest determinants, however this hypothesis was not confirmed. Our most robust finding showed younger age, higher MMSE scores at baseline and female gender as independent predictors of successful aging in this 16-year follow-up. Socioeconomic status,^4^ education^7^ and other social variables^6^ were longitudinally associated with SA in studies derived from developed countries and in two epidemiologic Brazilian cross-sectional investigations.^10^^,^ 11 Therefore we assume the various adverse conditions which older Brazilians could face such as health care barriers, poor infrastructure to deal with their needs, paucity of social network variables, and poor educational strategies impacting their chance of aging successfully. Despite the less robust association of income with SA found in the univariate analysis, none of these variables were predictors of SA in the multivariate model. As previously outlined, age has already been described as one of the strongest determinants of SA^3^^,^ 6 ^-^ 8 and we can suppose, even in less developed regions of the world, this biological variable has an inexorable effect upon health and death. The main causes of death found in our study (cardiovascular disease, cancer, pulmonary disease and stroke) were all age-related, strengthening this assumption. Furthermore, the impact of other variables on SA, such as income, could be lower in a longer follow-up in which not as many poorer people survive.

Female gender was also a predictor of SA in this sixteen-year cohort study, and this effect was retained in the age-adjusted model. The lower frequency of chronic and fatal diseases in women^24^ may be one of the reasons for this finding. In addition, women might have variables related to healthier life style and better qualitative aspects of social interactions during their lives which were not evaluated, but giving them a higher chance of aging successfully than men.

Another important finding of our study was the higher odds of individuals with greater MMSE baseline scores of being classified as SA in the sixteen-year follow-up. The better performance on this general cognitive screening tool could represent better cognitive reserve across lifespan. The better cognitive function could be the basis of many other healthier personal choices and strategies allowing individuals to age more successfully. On the other hand, those individuals that showed better cognitive scores in their sixties may be the “true” successful agers and the cognitive dimension may show superiority over the other aspects of SA such as the physical and the functional status. Additionally, good cognition could ensure better life engagement for older people, increasing the probability of aging successfully. However this hypothesis warrants further investigation. It is noteworthy that individuals classified as successful rather than normal agers at baseline did not keep this status in the follow-up. This result brings the view that aging is a dynamic process and subjects may change this classification throughout aging.

Limitations of our study should be mentioned. First, we had a significant number of drop-outs, which could affect the current findings. Despite different strategies to avoid losses during the follow-up (such as telephone contact, contact with neighbors, with potential relatives through telephone lists, major hospitals records, and official death register) we were not able to locate some participants, especially after the first follow-up interval. However, as previously mentioned, the comparison of the baseline main variables between the evaluated and the drop-out groups demonstrated no significant differences, except for age and education. This is an important problem in longitudinal studies with elderly people^25^ yet the 16-year assessment of community-dwelling older individuals in Southern Brazil is one of the major strengths of this study, furthering knowledge on aging characteristics of our population and helping inform future aging public politics in Brazil. Second, the definition of SA can raise special attention, because SA has been shown to be highly multidimensional and heterogeneous, especially in categorizing usual versus successful aging. We used the strict Rowe and Kahn’s definition, classifying few individuals as SA and probably having an effect in reducing the number of associated variables.

In conclusion, this cohort of community-dwelling older individuals in Southern Brazil highlighted the relevance of lower age, female gender and greater scores on MMSE as the strongest predictors of SA in a 16-year assessment. The impact of these variables was higher than socioeconomic or socio-environmental characteristics.
